# Supplementary material for: Genome-Wide Identification, Evolution, and Expression Analysis of the WD40 Subfamily in Oryza Genus
Source: Int J Mol Sci. 2023 Oct 30;24(21):15776. doi: 10.3390/ijms242115776 (PMC10648978; doi:10.3390/ijms242115776)
Supplement: Supplementary file 1 [file ijms-24-15776-s001.zip › SupplementFigures.pdf]

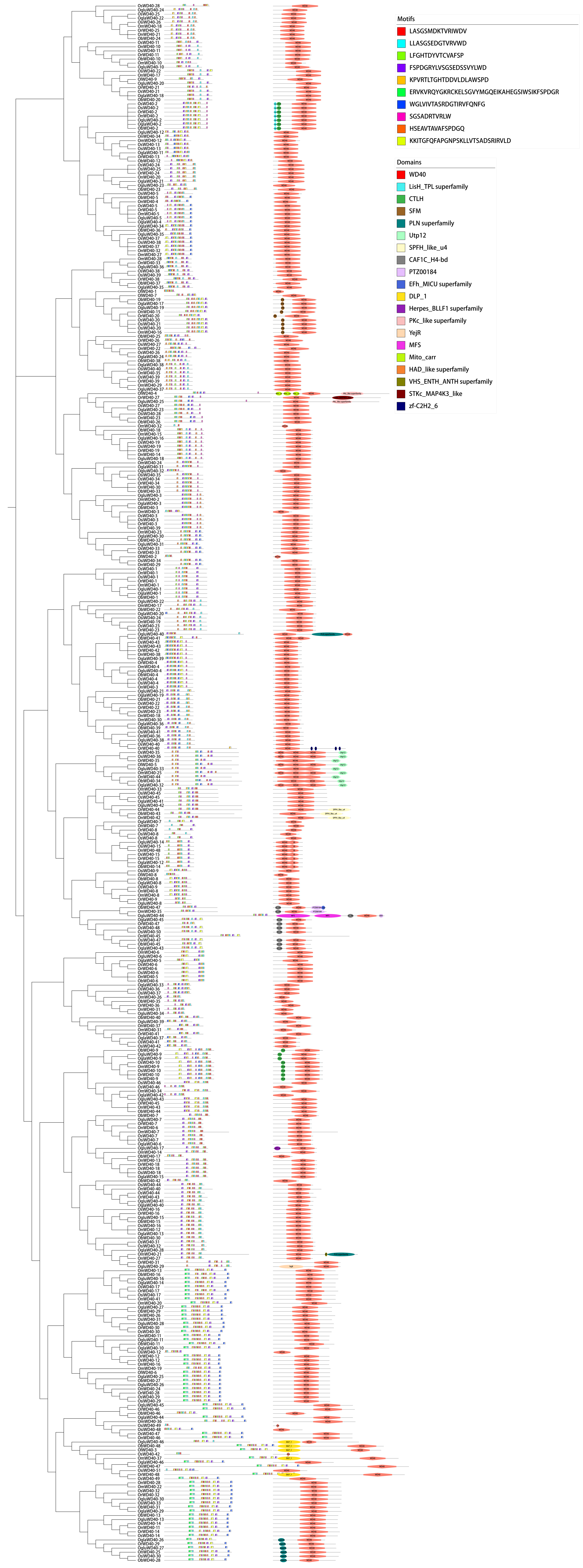

Figure S1. The conserved motifs and domains of WD40 subfamily in *Oryza* genus.

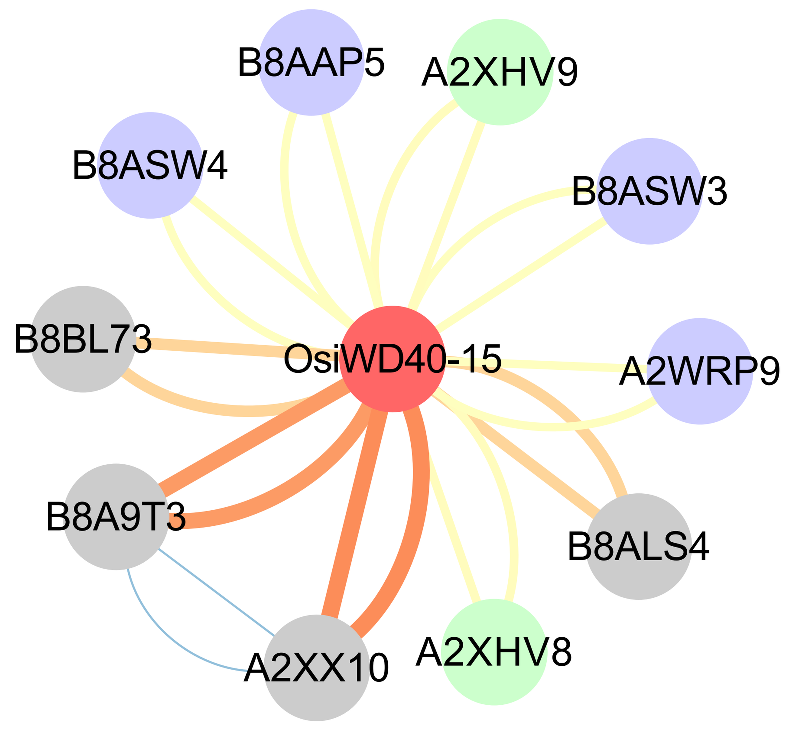

Figure S2. Protein-protein interaction network for OsiWD40s proteins. (a) The top 20 proteins in the center of the interactions network. (b) OsiWD40-15 protein-protein interaction analysis. Light purple represents bHLH protein, green represents MYB type protein, and gray represents other proteins
